# An assessment tool for the international healthy eating report card for preschool-aged children: a cross-cultural validation across Australia, Hong Kong, Singapore, and the United States

**DOI:** 10.3389/fnut.2024.1340007

**Published:** 2024-03-18

**Authors:** Alison Wing Lam Wan, Kevin Kien Hoa Chung, Jian-Bin Li, Shebe Siwei Xu, Derwin King Chung Chan

**Affiliations:** Department of Early Childhood Education, The Education University of Hong Kong, Tai Po, Hong Kong SAR, China

**Keywords:** scale development and testing, measurement invariance, healthy eating, report card, eating behaviours, family home food environment, preschool-aged children

## Abstract

**Objective:**

This study aimed to develop and validate a globally applicable assessment tool of the 43-item International Healthy Eating Report Card Scale (IHERCS) which was designed to assess preschool-aged children’s eating behaviours and family home food environments (FHFEs) across different cultural settings. In particular, we examined the factor structure, internal consistency and measurement invariance of the IHERCS across four cultural samples, including Australia, Hong Kong, Singapore, and the US. Convergent and discriminant validity were then conducted.

**Methods:**

In this cross-cultural study, a total of 2059 parent–child dyads from these four regions were recruited, and the parents were asked to complete the IHERCS. An exploratory structural equational modelling approach was employed to examine two higher-order factor models of children’s eating behaviours and FHFEs in the IHERCS and its cross-cultural measurement invariance.

**Results:**

The findings demonstrated robust factor structures of the scales of children’s eating behaviours and FHFEs in the IHERCS (i.e., CFI and TLI > 0.90; RMSEA and SRMR < 0.08) and an acceptable level of internal consistency (i.e., Cronbach’s α = 0.55–0.84). Full configural invariance and metric invariance were established across the four cultural contexts, but full scalar invariance was not achieved. Partial scalar invariance was found only in the scale of FHFEs. The convergent validity and discriminant validity were supported.

**Conclusion:**

Overall, the current findings provided preliminary support for the construct validity and measurement invariance of the IHERCS. It provides a reliable, valid and comprehensive assessment of eating behaviours and FHFEs among children in different cultural settings.

## Introduction

A region/country-level Report Card offers an effective approach that uses a traditional grading system (i.e., A+ to F) to comprehensively assess the prevalence of a particular health-related behaviour within a specific region or country (1). This approach is considered a succinct and informative tool because it has exhibited enormous success in increasing public awareness about the importance of targeted health behaviours (1). Not only does it stimulate broader discussion, but it also advocates for essential policy changes that further promote public health.

Given the success of the Physical Activity Report Card for Children and Youth (1–3), a recent study (4) has adopted its conceptual framework to develop a Healthy Eating Report Card for Pre-School Children in Hong Kong. This Healthy Eating Report Card incorporates various indicators related to children’s eating behaviours and family home food environments (FHFEs) to reveal the prevalence of healthy eating among pre-school children in Hong Kong. It also develops a parent-report questionnaire and corresponding benchmarks that are designed based on the guidelines and recommendations of the HKSAR Department of Health for healthy eating (4). However, applying such a region-specific Report Card to evaluate children’s healthy eating behaviours and FHFEs in other regions/countries could pose challenges because the guidelines and recommendations may vary across different geographical and cultural environments (5). To address this gap, it is highly important to develop an internationally applicable version of the Healthy Eating Report Card and its assessment tool. Additionally, the development stage of the measure of the Healthy Eating Report Card for Pre-School Children in Hong Kong does not involve any scale validation, which underscores another methodological limitation in terms of examining the psychometric properties of the assessment tool.

We, therefore, developed a new version of the International Healthy Eating Report Card for Preschool-aged Children and its globally applicable assessment tool of the International Healthy Eating Report Card Scale (IHERCS) based on the guidelines and recommendations of the global health authorities, including the World Health Organisation (6–8), the European Food Safety Authority (9), the American Academy of Pediatrics (10), the Centers for Disease Control and Prevention (11) and the relevant literature (12–18). A significant change in the Report Card involved replacing a prior indicator “Avoidance of Unhealthy Foods” with “Home Healthier Food Availability and Accessibility” by considering the pivotal role of home food availability and accessibility in shaping FHFEs and influencing children’s eating behaviours (19, 20). In addition, the IHERCS expanded from the 21-item parent-report measure in a prior study (4) to include a total of 43 items. The additional items allowed for a more comprehensive assessment of children’s healthy eating behaviours (e.g., children’s unhealthy snack consumption) and FHFEs (e.g., both healthy and unhealthy food availability and accessibility within the home). All the dimensions of IHERCS aligned with the International Healthy Eating Report Card (i.e., Indicators of Children’s Eating Behaviours: (1) Children’s Dietary Patterns and (2) Children’s Mealtime Behaviours and Indicators of FHFEs: (3) Parental Food Choices and Preparation, (4) Home Healthier Food Availability and Accessibility and (5) Family Mealtime Environments). The assessment tool aimed to assess the extent to which preschool-aged children in different cultural contexts adhere to the standards and recommendations of the global health authorities for healthy eating behaviours and get involved in favourable FHFEs. Both English and Chinese versions of the IHERCS are in Appendices A and B.

To the best of our knowledge, there is currently no existing single measure that comprehensively covers all aspects of children’s healthy eating patterns as assessed by the IHERCS. Existing methods for evaluating children’s dietary patterns typically include dietary recalls, dietary records and food checklists (e.g., the Food Frequency Questionnaire) (21). While these assessments provide detailed information on dietary intake, they lack an explicit design for assessing the extent to which children adhere to global guidelines for healthy eating. Parent-reported measures, such as the Behavioural Pediatric Feeding Assessment Scale (BPFAS) and About Your Child’s Eating-Revised, are well-established scales that assess the mealtime behaviours of children (22). However, these measures focus on evaluating the parents’ feeding strategies and feelings about mealtimes rather than solely assessing the frequency of mealtime behaviours for typically developing children. Additionally, there is a lack of established assessment tools designed to evaluate the frequency with which parents adopt healthy food choices and food preparation practices for their children. Validated questionnaires that concurrently assess both the availability and accessibility of healthy and unhealthy food within the household are scarce (20, 23–27), and they do not account for the availability and accessibility of plain water (28). Another validated measure, the Meals in Our Household Questionnaire (MOH), assesses the frequency of structured family mealtime environments, but it does not account for the presence of common distracting objects (e.g., toys, phones and tablets) during mealtime (29).

To address the limitations of the former assessment tools, this study aimed to conduct a cross-cultural study to validate the assessment tool of the IHERCS. In particular, we first examined the factor structure, internal consistency and measurement invariance of the IHERCS across four cultural groups [i.e., Australia, Hong Kong, Singapore and the United States (US)]. The BPFAS and the MOH were then used to examine the convergent and discriminant validity of the IHERCS. The selection of these four countries for this study was based on their representation of a diverse range of developed countries/regions with distinct cultural contexts. Australia and the US are the Western countries representative of the North America and Oceania continents, respectively, while Hong Kong and Singapore are important cities located in Asia and Southeast Asia regions. These four regions are well recognised as developed regions with comparable socioeconomic characteristics [i.e., ranked in a very high Human Development Index category (HDI ≥ 0.9)], and their samples should reflect a diversity of dietary patterns in different cultures (30).

Based on the conceptual framework of the initial Healthy Eating Report Card (4), it was hypothesised that:

*H1*: The factor structure of the IHERCS would be comparable to that of the initial Health Eating Report Card (4), and would yield acceptable model fit indices. In particular, we expected the IHERCS would have a total of eleven factors under two higher-order factors of children’s eating behaviours and FHFEs. Three factors (i.e., food variety, low consumption of unhealthy foods, and mealtime behaviours) would reflect children’s eating behaviours, and eight factors (i.e., parental food choices, parental food preparation, healthy food availability, unhealthy food availability, healthy food accessibility, unhealthy food accessibility, reduction in mealtime distractions, and structure of family mealtime) would reflect children’s FHFEs. This factor structure would be supported by acceptable model fit and internal reliability of the factors.

*H2*: The factor structure of the IHERCS as proposed in H1 would exhibit psychometric invariance across four cultural contexts.

*H3*: The convergent validity of the IHERCS (i.e., factors of mealtime behaviours and structure of family mealtime) would be evident by significant and positive relationships between the factors of the IHERCS and the scores of other validated scales of children’s healthy eating (i.e., subscales in the BPFAS and MOH).

*H4*: The discriminant validity of the IHERCS would be revealed by children’s eating behaviours and FHFEs of the IHERCS being statistically independent from the subscales of the MOH and BPFAS.

## Method

### Participants

Data collection was conducted in early December 2022. We used the survey panel service of Qualtrics to recruit participants residing in Australia, Hong Kong, Singapore and the US. The inclusion criteria for eligible participants were considered as follows: (i) participants must reside in Australia, Hong Kong, Singapore or the US, (ii) parents must have at least one child, (iii) the age range of the oldest child among their children must be between 2 and 6 years old and (iv) parents are able to read Chinese or English for questionnaire completion. Children with any medical conditions were not included in the present study. We planned to recruit more than 500 parent–child dyads from each region, as the sample size was comparable to that of the initial study of the Healthy Eating Report Card (4). Finally, we recruited a total of 2059 parent–child dyads from Australia (*n* = 500), Hong Kong (*n* = 552), Singapore (*n* = 507), and the US (*n* = 500) who fulfilled the inclusion/exclusion criteria of our study. Among the parent respondents (mean age = 36; SD = 6.99; range = 21–71 years), 60.95% were mothers, 37.49% were fathers, and 1.56% were listed as other guardians. The child participants in the study had a mean age of 4.56 (SD = 1.32), and 49.9% were boys. Demographic details of the participants from each cultural context are presented in Table 1.

**Table 1:** Demographic characteristics of the samples.

	Whole sample (*n* = 2059)		Australia (*n* = 500)		Hong Kong (*n* = 552)		Singapore (*n* = 507)		The United States (*n* = 500)	
	*n*	%	*n*	%	*n*	%	*n*	%	*n*	%
**Children**										
Mean age (SD)	*M* = 4.56 (SD = 1.32)		*M* = 4.51 (SD = 1.36)		*M* = 4.53 (SD = 1.26)		*M* = 4.65 (SD = 1.30)		*M* = 4.57 (SD = 1.34)	
Genders										
Boys	1,027	49.88	246	49.2	282	51.09	251	49.51	248	49.6
Girls	1,032	50.12	254	50.8	270	48.91	256	50.49	252	50.4
**Parents/guardians**										
Mean age (SD)	*M* = 36 (SD = 6.99)		*M* = 35.02 (SD = 7.01)		*M* = 37.59 (SD = 6.63)		*M* = 36.48 (SD = 6.35)		*M* = 34.69 (SD = 7.58)	
Respondents										
Father	772	37.49	120	24	245	44.4	267	52.66	140	28
Mother	1,255	60.95	375	75	299	54.2	233	45.96	348	69.6
Others	32	1.55	5	1	8	1.4	7	1.38	12	2.4
Marital status										
Married	1701	82.61	393	78.6	534	96.74	486	95.86	288	57.6
Widowed	11	0.53	3	0.6	0	0	1	0.2	7	1.4
Divorced	69	3.35	11	2.2	7	1.27	5	0.99	46	9.2
Separated	38	1.85	20	4	2	0.36	2	0.39	14	2.8
Single	240	11.66	73	14.6	9	1.63	13	2.56	145	29
Education level										
Primary school	58	2.82	27	5.4	4	0.72	10	1.97	17	3.4
Secondary school	840	40.8	238	47.6	173	31.34	124	24.46	305	61
University	1,161	56.39	235	47	375	67.93	373	73.57	178	35.6

### Procedure

The research protocol for this study was approved by the Human Research Ethics Committee of the first author’s institution (Ref. no. 2022-2023-0033). This cross-cultural study in which participants from the four cultural contexts were asked to complete a questionnaire comprising the IHERCS and other validated scales related to children’s mealtime behaviours and family mealtime environment. After signing the online consent form to indicate that they agreed to participate in the study, the parent participants were instructed to complete an online self-report questionnaire once. The questionnaire could be completed jointly by the parent and caregiver who primarily take care of the children.

### Measures

#### Demographic factors

Demographic questions were included to assess respondents’ personal information, such as gender, age, respondent to questionnaire (i.e., mother, father or other guardian) and education level. In addition, their children’s personal information was obtained, including gender and date of birth.

#### IHERCS

The IHERCS included two scales with a total of 43 items to assess children’s eating behaviours and FHFEs. The scale of children’s eating behaviours consists of 13 items to assess three dimensions: children’s food variety, low consumption of unhealthy foods and children’s mealtime behaviours. Three additional questions were incorporated to assess certain dietary patterns (e.g., breakfast consumption). The scale of FHFEs consists of 27 items to assess eight dimensions: parental food choices, parental food preparation, healthy food availability, unhealthy food availability, healthy food accessibility, unhealthy food accessibility, reduction in mealtime distractions, and structure of family mealtime. Items corresponding to the latent variable are presented in Appendix C. Respondents responded to the items using the open-ended question, multiple choice question and 5-point Likert scale [i.e., ranging from 1 (Never) to 5 (Daily)].

#### BPFAS

We used the BPFAS to provide an alternative assessment of the frequency of particular mealtime behaviours of children. BPFAS has been widely used in different countries and populations and regarded as a well-established measure with relatively high reliability (within the acceptable to excellent range) and robust psychometric properties (22, 31). It consists of 25 items to assess the frequency of children’s mealtime behaviours with a 5-point Likert scale ranging from 1 (Never) to 5 (Always). In line with the dimension of mealtime behaviours in IHERCS, we reverse-scored negative items in BPFAS with higher scores representing more appropriate mealtime behaviours in children.

#### MOH

We adopted the MOH to provide an alternative assessment of the frequency of children involved in certain structured family mealtime environments. While the MOH has only been used in a single population sample, it has demonstrated an acceptable level of internal consistency, excellent test–retest reliability and robust construct validity (22, 29). It consists of 10 items to assess children’s mealtime environments within their households with a 4-point Likert scale ranging from 1 (Never) to 4 (Almost Always).

### Statistical analyses

All analyses in the present study were conducted with SPSS for descriptive analyses and MPlus 8 (Muthén and Muthén, 1998–2017) for conducting exploratory structural equational modelling (ESEM) to examine two higher-order factor models of children’s eating behaviours and FHFEs. Considering the results of Mardia’s multivariate normality test indicating a violation of the multivariate normality assumption (Sk_M_ = 0.81, *p* < 0.001, Ku_M_ = 13.69, *p* < 0.001; Sk_M_ = 7.14, *p* < 0.001, Ku_M_ = 95.83, *p* < 0.001), the maximum likelihood with robust standard errors (MLR) was employed to handle the non-normality (32). The small proportion of missing values (0.2%) in the dataset was handled using the full information maximum likelihood.

#### Factor structure and internal consistency

An ESEM approach was adopted to assess the factor structure of the assessment tool across four cultural contexts. We used multiple indicators to assess the goodness of fit of the proposed models with CFI and TLI values exceeding 0.90 and RMSEA and SRMR values below 0.08 (33). For internal consistency, Cronbach’s alphas for all factors across each of the cultural contexts were calculated. Due to the limited number of items in the latent factor, a minimally acceptable cut-off value greater than 0.50 was used as a criterion (34, 35).

#### Measurement invariance by cultural context

Multigroup ESEM was employed to test for measurement invariance. The typical steps for testing measurement invariance were followed, including configural invariance, metric invariance and scalar invariance across four cultural contexts. In the first step, we tested the configural invariance model without imposing any equality constraint by following cut-off criteria for adequate model fit (CFI > 0.90, TLI > 0.90, SRMR < 0.08, RMSEA < 0.08) (33). If the configural invariance model was supported, we constrained the factor loadings to be equivalent in the four cultural contexts for testing the metric invariance model and subsequently testing scalar invariance by constraining the intercepts to be equivalent. Criteria for model fit changes included a decrease in CFI ≤ 0.010 and an increase in RMSEA ≤ 0.015 and SRMR of ≤ 0.030 (for metric invariance) or ≤ 0.010 (for scalar invariance) (36, 37). If metric or scalar invariance was not supported, a partial invariance model would be tested by releasing equality constraints on one or more loadings or intercepts to achieve an acceptable goodness of fit.

#### Convergent and discriminant validity

Pearson’s correlation analysis was employed to assess the convergent validity of mealtime behaviours and the structure of family mealtime. A correlation coefficient of 0.30 or higher indicated an acceptable level of convergent validity (38). For discriminant validity, we estimated the shared variance between the factors in the IHERCS and the validated scales as well as the average variance extracted (AVE) for each of their factors. The AVE was calculated by summing the squared factor loadings and dividing it by the number of items. Discriminant validity was established when the AVE was greater than the shared variance between the constructs (33).

## Results

### Factor structure and internal consistency

We initially tested the factor structure of both scales of children’s eating behaviours and FHFEs across four cultural contexts. ESEM yielded a three-factor model for the scale of children’s eating behaviours and an eight-factor model for the scale of FHFEs with a satisfactory fit (χ^2^ = 258.52 (*df* = 41), CFI = 0.96, RMSEA = 0.05 [90% CI = 0.05 to 0.06], and SRMR = 0.03 and χ^2^ = 660.64 (*df* = 161), CFI = 0.97, RMSEA = 0.04 [90% CI = 0.04 to 0.04], and SRMR = 0.02). Values of Cronbach’s alpha were acceptable, ranging from 0.55 to 0.84. Model fit indexes, Cronbach’s alpha coefficients and the range of item loadings to target factors for each cultural context are presented in Tables 2–4.

**Table 2:** Factor structure of the IHERCS using exploratory structural equation modelling.

	χ^2^	df	*p*	CFI	TLI	RMSEA [90% CI]	SRMR
**Scale of children’s eating behaviours**							
Whole by cultural contexts	258.516	41	< 0.001	0.959	0.921	0.051 [0.045, 0.057]	0.025
Australia	88.456	41	< 0.001	0.961	0.925	0.048 [0.034, 0.062]	0.033
Hong Kong	96.691	41	< 0.001	0.964	0.931	0.050 [0.037, 0.062]	0.029
Singapore	95.128	41	< 0.001	0.964	0.931	0.051 [0.038, 0.065]	0.032
The United States	64.149	41	< 0.001	0.980	0.961	0.034 [0.016, 0.049]	0.032
**Scale of family home food environments**							
Whole by cultural contexts	660.639	161	< 0.001	0.974	0.944	0.039 [0.036, 0.042]	0.016
Australia	350.742	161	< 0.001	0.963	0.918	0.049 [0.042, 0.055]	0.023
Hong Kong	375.249	161	< 0.001	0.960	0.914	0.049 [0.043, 0.056]	0.019
Singapore	276.669	161	< 0.001	0.977	0.951	0.038 [0.030, 0.045]	0.017
The United States	357.926	161	< 0.001	0.956	0.903	0.049 [0.043, 0.056]	0.023

CFI = comparative fit index; TLI = Tucker-Lewis index; RMSEA = root mean square error of approximation; 90% CI = 90% confidence interval of the RMSEA; SRMR = standardised root mean square residual.

**Table 3:** Correlation matrix, factor loadings and validity indexes of the scale of children’s eating behaviours in the IHERCS.

	1	2	3	4	5
1. Dietary variety	−				
2. Low consumption of unhealthy food	0.183^**^	−			
3. Mealtime behaviours	0.213^**^	0.257^**^	−		
4. BPFAS	0.294^**^	0.282^**^	0.723^**^	−	
5. MOH	0.186^**^	0.101^**^	0.109^**^	0.377^**^	−
Range of item loadings to target factors (ESEM)	0.664–0.858 [0.481–0.952]	0.514–0.980 [0.396–0.931]	0.601–0.748 [0.540–0.770]	N/A	N/A
Range of mean	0.83 [0.68–1.04]	1.32 [0.96–1.49]	23.19 [22.6–23.85]	N/A	N/A
Range of SD	0.98 [0.93–1.02]	1.03 [0.95–1.03]	5.4 [5.39–5.76]	N/A	N/A
Cronbach’s Alpha	0.613 [0.585–0.648]	0.605 [0.564–0.644]	0.843 [0.839–0.864]	N/A	N/A
AVE	0.565	0.504	0.520	N/A	N/A
Shared variance (*R*)^2^	0.035	0.010	0.012	N/A	N/A

BPFAS = subscale for assessing the frequency of mealtime behaviours of children in the BPFAS; MOH = subscale for assessing structure of family meals in Meals in our Household Questionnaire; Range of item loadings to target factors (ESEM) = item loadings (exploratory structural equation modelling) on the whole sample [range across cultural contexts]; Cronbach’s alpha = Cronbach’s alpha on the whole sample [range across cultural contexts]; Range of mean = the mean score on the whole sample [range across cultural contexts]; Range of SD = standard deviation on the whole sample [range across cultural contexts]; AVE = average variance extracted. *p < 0.05. **p < 0.01.

**Table 4:** Correlation matrix, factor loadings and validity indexes of the scale of family home food environments in the IHERCS.

	1	2	3	4	5	6	7	8	9	10
1. Parental food choices	−									
2. Parental food preparation	−0.172^**^	−								
3. Availability of healthy food	0.276^**^	0.136^**^	−							
4. Availability of unhealthy food	−0.099^**^	−0.316^**^	0.122^**^	−						
5. Accessibility of healthy food	0.268^**^	−0.021	0.589^**^	0.062^**^	−					
6. Accessibility of unhealthy food	−0.052^*^	−0.437^**^	−0.158^**^	0.518^**^	0.097^**^	−				
7. Reduction in mealtime distraction	−0.007	0.395^**^	0.252^**^	−0.291^**^	0.117^**^	−0.424^**^	−			
8. Mealtime environment	0.295^**^	0.088^**^	0.624^**^	0.041	0.406^**^	−0.171^**^	0.257^**^	−		
9. BPFAS	−0.001	0.335^**^	0.408^**^	−0.193^**^	0.248^**^	−0.340^**^	0.485^**^	0.409^**^	−	
10. MOH	0.269^**^	0.056^*^	0.618^**^	0.074^**^	0.426^**^	−0.137^**^	0.243^**^	0.816^**^	0.377^**^	−
Range of item loadings to target factors (ESEM)	0.476–0.743 [0.314–0.764]	0.395–0.583 [0.343–0.786]	0.387–0.720 [0.339–0.844]	0.613–0.718 [0.390–0.722]	0.624–0.736 [0.502–0.862]	0.567–0.776 [0.433–0.720]	0.503–0.920 [0.500–0.865]	0.521–0.763 [0.392–0.871]	N/A	N/A
Range of mean	19.03 [18.23−19.46]	9.94 [9.48−10.29]	12.66 [11.82−13.52]	6.07 [5.79–6.52]	11.37 [10.48−12.42]	5.17 [4.8–5.56]	10.57 [10.24−11.03]	19.88 [18.82−20.71]	N/A	N/A
Range of SD	4.2 [3.89–4.41]	2.27 [1.98–2.44]	2.42 [2.17–2.54]	1.96 [1.9–2.03]	2.68 [2.41–2.67]	2.19 [1.99–2.27]	2.88 [2.63–3.05]	3.59 [3.34–3.76]	N/A	N/A
Cronbach’s alpha	0.792 [0.769–0.807]	0.550 [0.524–0.607]	0.820 [0.779–0.838]	0.632 [0.563–0.734]	0.712 [0.647–0.724]	0.755 [0.712–0.770]	0.680 [0.654–0.730]	0.783 [0.701–0.825]	N/A	N/A
AVE	0.491	0.494	0.717	0.674	0.674	0.784	0.568	0.533	N/A	N/A
Shared variance (*R*)^2^	0.004	0.136	0.000	0.078	0.005	0.122	0.212	0.057	N/A	N/A

BPFAS = subscale for assessing the frequency of mealtime behaviours of children in the BPFAS; MOH = subscale for assessing structure of family meals in Meals in our Household Questionnaire; Range of item loadings to target factors (ESEM) = item loadings (exploratory structural equation modelling) on the whole sample [range across cultural contexts]; Cronbach’s alpha = Cronbach’s alpha on the whole sample [range across cultural contexts]; Range of mean = the mean score on the whole sample [range across cultural contexts]; Range of SD = standard deviation on the whole sample [range across cultural contexts]; AVE = average variance extracted.

*p < 0.05. **p < 0.01.

### Measurement invariance between cultural contexts

Both configural invariance models of the scale of children’s eating behaviours (Model 1a) and the scale of FHFEs (Model 1b) yielded an acceptable fit to the data, χ^2^ = 476.83 (*df* = 168), CFI = 0.95, RMSEA = 0.06 [90% CI = 0.05 to 0.07], and SRMR = 0.03 and χ^2^ = 1600.35 (*df* = 652), CFI = 0.94, RMSEA = 0.05 [90% CI = 0.05 to 0.06], and SRMR = 0.02, indicating that the model exhibited configural equivalence across different cultural contexts. Second, the test of metric invariance showed that Model 2a and Model 2b were not significantly different from Models 1a and Model 1b (ΔCFI ≤ 0.010, ΔRMSEA ≤ 0.015, ΔSRMR ≤ 0.030), indicating support for full metric invariance. The scalar invariance models of both scales (Models 3a and 3b) were tested by imposing equality constraints on all item intercepts. The results showed that neither scale achieved scalar invariance (ΔCFI ≥ 0.010, ΔRMSEA ≥ 0.015, ΔSRMR ≥ 0.010).

Due to the lack of scalar invariance, partial scalar invariance of both scales was conducted to relax noninvariant items across all cultural contexts (Models 4a and 4b). In Model 2a, items 1.1 and 1.4 were found to have the smallest residual variance in the metric invariance model, while items 3.1, 3.2, 3.3, 4.1.b and 5.1 had the smallest residual variance in Model 2b. After relaxing the equality constraints, partial scalar invariance was supported in Model 4b (ΔCFI ≤ 0.010, ΔRMSEA ≤ 0.015, ΔSRMR ≤ 0.010), but rejected in Model 4a (ΔCFI ≥ 0.010, ΔRMSEA ≥ 0.015, ΔSRMR ≥ 0.010). The results are displayed in Table 5.

**Table 5:** Measurement invariance of the IHERCS across four cultural contexts.

MG-ESEM comparison	*N*	χ^2^ (*df*)	*p*	CFI	RMSEA [90% CI]	SRMR	*M* comparison	ΔCFI	∆RMSEA	∆SRMR
**Scale of children’s eating behaviours**										
M1a: configural invariance	2059	476.828 (168)	< 0.001	0.953	0.060 [0.053, 0.066]	0.027	−	−	−	−
M2a: metric invariance	2059	607.913 (258)	< 0.001	0.946	0.051 [0.046, 0.057]	0.044	M2a − M1a	−0.007	−0.009	0.017
M3a: scalar invariance	2059	1029.767 (288)	< 0.001	0.886	0.071 [0.066, 0.075]	0.057	M3a − M2a	−0.060	0.020	0.013
M4a: partial scalar invariance	2059	1000.527 (282)	< 0.001	0.890	0.070 [0.066, 0.075]	0.055	M4a − M2a	−0.056	0.019	0.011
**Scale of FHFEs**										
M1b: configural invariance	2059	1600.347 (652)	< 0.001	0.942	0.053 [0.050, 0.056]	0.021	−	−	−	−
M2b: metric invariance	2059	2207.656 (1108)	< 0.001	0.932	0.044 [0.041, 0.047]	0.044	M2b − M1b	−0.010	−0.009	0.023
M3b: scalar invariance	2059	2589.144 (1165)	< 0.001	0.912	0.049 [0.046, 0.051]	0.048	M3b − M2b	−0.020	0.005	0.004
M4b: partial scalar invariance	2059	2425.322 (1150)	< 0.001	0.922	0.046 [0.044, 0.049]	0.048	M4b − M2b	−0.010	0.002	0.004

MG-ESEM = multigroup exploratory structural equation modelling; CFI = comparative fit index; RMSEA = root mean square error of approximation; 90% CI = 90% confidence interval of the RMSEA; SRMR = standardised root mean square error of approximation; ΔCFI = change in CFI; ΔRMSEA = change in RMSEA; Δ SRMR = change in SRMR.

### Convergent and discriminant validity

For convergent validity, there was a significant positive relationship between the score of the dimension of mealtime behaviours and the BPFAS (*r* = 0.72, *p* < 0.01). In addition, the score of the dimension of structure of family mealtime showed a significant positive relationship with the MOH (*r* = 0.82, *p* < 0.01). For discriminant validity, the AVEs for the variables in the scale of children’s eating behaviours (range: 0.52 to 0.57) were higher than the shared variances with the MOH (range: 0.01 to 0.04). Similarly, the AVEs for the variables in the scale of FHFEs (range: 0.49 to 0.78) were higher than the shared variance with the BPFAS (range: 0.00 to 0.21). The correlation matrix and validity indexes are presented in Tables 3 and 4.

## Discussion

The current study aimed to examine the psychometric properties and measurement invariance of the IHERCS among pre-school aged children from four cultural samples in Asian and Western societies (i.e., Australia, Hong Kong, Singapore and the US). The IHERCS included the comprehensive assessment of both children’s eating behaviours and FHFEs. It was designed to examine the extent to which preschool-aged children adhere to the standards and recommendations of the global health authorities for healthy eating. This tool may reveal the prevalence status of healthy eating in preschool-aged children from different cities/countries and may have the potential to increase public awareness of healthy eating practices among young children. In general, as supported by our findings, the IHERCS demonstrated its value as a comprehensive, valid and culturally relevant tool for assessing children’s eating behaviours and FHFEs.

The findings from over two thousand preschool-aged children provided robust support for the construct validity (i.e., established through factor structure, convergent validity, discriminant validity) and measurement invariance. The findings were in line with our hypothesis that supported the robustness of the construct validity of the IHERCS. Moreover, we successfully established full configural invariance and metric invariance for both the scale of children’s eating behaviours and the scale of FHFEs across the four cultural contexts indicating that the model structure was invariant across samples. However, full scalar invariance could not be established for either of these scales. While partial scalar invariance was achieved for the scale of FHFEs after relaxing specific constrained parameters, the same level of scalar invariance was not confirmed for the scale of children’s eating behaviours. This was potentially attributed to cultural differences in the interpretation and scoring of the items of children’s eating behaviours (39).

The IHERCS represented an extended version of the assessment tool of the Healthy Eating Report Card for Pre-School Children in Hong Kong used in the prior study (4). It is the first validated parent-reported measure designed to comprehensively assess a wide range of children’s eating behaviours and FHFEs. Compared to other validated assessment tools for children’s dietary patterns and mealtime behaviours (e.g., the Food Frequency Questionnaire and the BPFAS), the scale of children’s eating behaviours in the IHERCS is relatively shorter and simpler, but the sixteen items of the scale can still provide a comprehensive assessment of preschool children’s adherence to dietary patterns and the frequency of mealtime behaviours. This makes the IHERCS particularly suitable for large-scale studies (21, 31), as it may reduce the response burden on participants while efficiently assessing the eating behaviour of young children. Additionally, the IHERCS may also be utilised in evaluating the effectiveness of interventions or public health policy that aims to promote preschool children’s healthy eating patterns.

The scale of FHFEs in the IHERCS extensively assesses various family-related factors that may influence children’s eating behaviours, including parents’ personal and behavioural factors (i.e., parental food choices and preparation), physical environment factors (i.e., home food availability and accessibility), and family social environmental factors (i.e., family mealtime environments) (20). By initially assessing the frequency with which parents make healthy food choices and adopt healthy cooking practices for their children, the IHERCS offers new insights into the evaluation of children’s FHFEs by taking parental factors of children’s eating behaviours into account. Moreover, the IHERCS provides a concise instrument for assessing the availability and accessibility of both healthy and unhealthy food within the household, including the assessment for plain water. Unlike other assessments (e.g., the MOH) that solely assess TV-related distraction during mealtime, the IHERCS considers other common distracting objects during mealtime (e.g., phones and toys) (29). As such, this comprehensive approach in the scale of FHFEs allows for a holistic evaluation of the parental and family-related factors of children’s eating behaviours.

In sum, our study presents the first cross-cultural psychometric validation study of the IHERCS, which can be considered an international tool to assess the eating behaviours and FHFEs of preschool-aged children that was designed based on the guidelines of global health authorities for healthy eating. The positive findings from our study supported the use of the IHERCS as a reliable assessment tool for revealing the prevalence of healthy eating among children in different cultural settings. Despite the strengths and novelty of our study, we have to acknowledge a few study limitations. First, with a cross-sectional design, our study could not reveal the temporal stability of children’s eating behaviours and FHFEs over time, and the test–retest reliability of the IHERCS remains unclear. Additionally, our study did not include any measures of health-related outcomes (e.g., self-rated health, quality of life and mental health), so we were unable to examine the criterion validity and predictive validity of the IHERCS. Future studies should adopt a longitudinal design to examine the temporal stability of the IHERCS, and the extent to which the scale is predictive of other behavioural or health outcomes over time (40). Second, the study only relied on parent-reported measures to assess children’s eating behaviours and FHFEs, so the responses could be susceptible to social desirability and self-serving biases. The homogeneous Likert-scale type of responses in our survey-based study could induce common method variance due to general response tendency, resulting in possibly inflating inter-factor correlations and internal reliability of the IHERCS within our study (41, 42). Future studies may consider completing the IHERCS in an objective manner by introducing independent assessors, observational techniques (e.g., dietary recalls), and verification by dietitians to enhance the accuracy and objectivity of responses.

## Conclusion

The present study developed and evaluated the construct validity (i.e., factor structure, convergent and discriminant validity), and measurement invariance of the IHERCS across four cultural settings (i.e., Australia, Hong Kong, Singapore and the US). The IHERCS was designed to align with the indicators of the International Healthy Eating Report Card for Preschool-Aged Children, with the aim of providing a comprehensive assessment of eating behaviours and FHFEs among preschool-aged children in different cultural settings. The current findings provided preliminary support for the factor structure and internal consistency. Configural invariance and metric invariance were supported, but only partial scalar invariance was found in the scale of FHFEs. The IHERCS also met the convergent and discriminant validity. We hope the initial evidence of the psychometric properties of the IHERCS will stimulate future studies in applying this report card tool in assessing a wide range of aspects of eating behaviours and FHFEs across different cultures.

## Data availability statement

The raw data supporting the conclusions of this article will be made available by the authors, without undue reservation.

## Ethics statement

The studies involving humans were approved by Human Research Ethics Committee of the Education University of Hong Kong. The studies were conducted in accordance with the local legislation and institutional requirements. The participants provided their written informed consent to participate in this study.

## Author contributions

AW: Conceptualization, Data curation, Formal analysis, Investigation, Methodology, Project administration, Validation, Writing – original draft, Writing – review & editing. KC: Conceptualization, Investigation, Project administration, Supervision, Writing – review & editing. J-BL: Conceptualization, Investigation, Project administration, Supervision, Writing – review & editing. SX: Formal analysis, Investigation, Methodology, Writing – review & editing, Validation. DC: Conceptualization, Funding acquisition, Investigation, Project administration, Resources, Supervision, Validation, Writing – review & editing.
