# Supplementary material for: An assessment tool for the international healthy eating report card for preschool-aged children: a cross-cultural validation across Australia, Hong Kong, Singapore, and the United States
Source: Front Nutr. 2024 Mar 18;11:1340007. doi: 10.3389/fnut.2024.1340007 (PMC10982429; doi:10.3389/fnut.2024.1340007)
Supplement: Supplementary file 1 [file Data_Sheet_1.pdf]

Appendix A (online supplementary material)

<u>The Assessment Scales of the International Healthy Eating Report Card for Preschool-Aged Children (English Version)</u>

Part 1: Understanding your child's dietary patterns

Item		
1.1	In the past 7 days, how many days did your child eat the following types of food?	
	**a. Starchy staples**<br><br>Cereals (corn/maise, rice, wheat, or any other grains or foods made from these (e.g., bread, noodles, porridge, breakfast cereal or other grain products) and white tubers and roots (e.g., white potatoes, white yam, white cassava, or other foods made from roots)<br>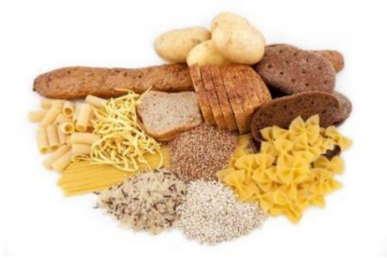	**______day(s)**
	**b. Vegetables**<br><br>Leafy greens (e.g., spinach, lettuces, seaweeds) and other vegetables (e.g., onions, eggplants, bell peppers, mushrooms, tomatoes), **<u>excluding starchy roots and tubers (e.g., potatoes, sweet potatoes) and pulses (e.g., lentils, beans)</u>**<br>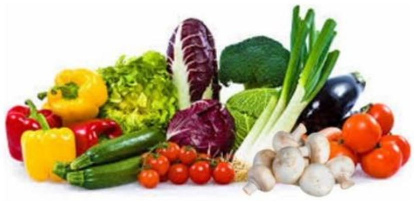	**______day(s)**
	**c. Fresh fruits**<br><br>e.g., Apples, cantaloupes, strawberries, grapes, watermelons, plums, etc.<br>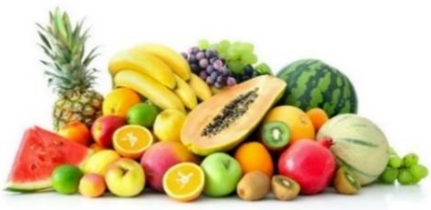	**______day(s)**

	**d. Meat, poultry, fish, egg, legumes, nuts and seeds**<br><br>e.g., Beef, pork, chicken, fish, shrimp, crabs, chicken eggs, chickpeas, lentils, tofu, almonds, chia seeds, pumpkin seeds	**______day(s)**
	**e. Dairy and alternatives**<br><br>e.g., Cow's milk, cheese, yogurt, calcium-fortified soy milk	**______day(s)**
	* A respondent answered 7 days per week for each food group indicating that a child eats a variety of foods from each of the five main food groups daily. In this case, it is scored as "yes=1".	

1.2 In the past 7 days, on average how many cups of vegetables did your child eat each day?

<u>e.g., Leafy greens (e.g., spinach, lettuces, seaweeds) or other vegetables (e.g., onions, eggplants, bell peppers, mushrooms, tomatoes),</u> **<u>excluding starchy roots and tubers (e.g., potatoes, sweet potatoes) and pulses (e.g., lentils, beans)</u>**

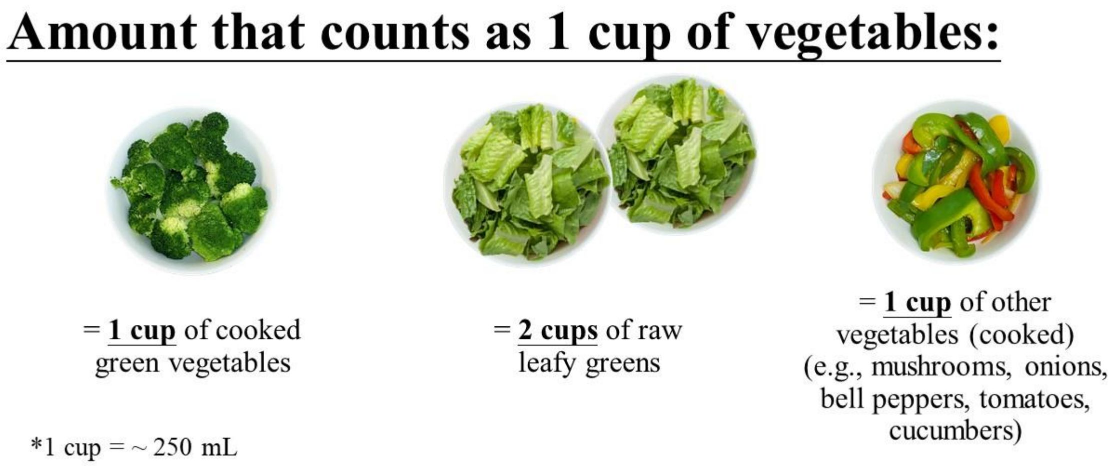

An average of ______________cup (s) per day

Your answer can be in whole or half cups (e.g., 0.5, 1, 1.5 etc.)

* A respondent answered ≥1 cup/day for children aged 2-3-year-old or ≥1.5 cup/day for children aged 4-8-year-old indicating that a child has an adequate intake of vegetable daily. In this case, it is scored as "yes=1".

1.3	In the past 7 days, on average how many cups of fresh fruit did your child eat each day?<br>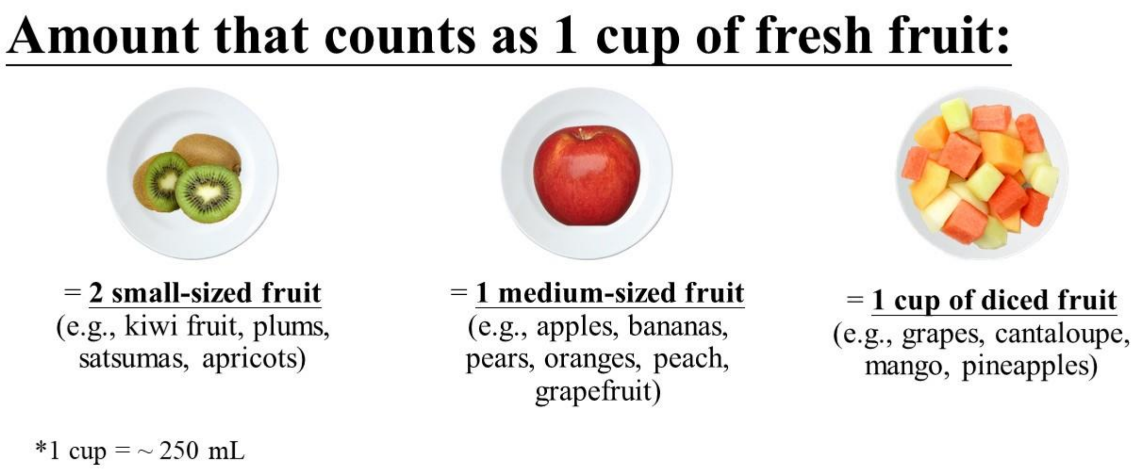<br><br>**An average of ______________cup (s) per day**<br>Your answer can be in whole or half cups (e.g., 0.5, 1, 1.5 etc.)<br>* A respondent answered ≥1 cup/day for children aged 2-3-year-old or ≥1.5 cup/day for children aged 4-8-year-old indicating that a child has an adequate intake of fruit daily. In this case, it is scored as "yes=1".
1.4	In the past 7 days, did your child eat snacks between meals? (Please also count snacks consumed at school)<br>□ Yes □ No<br>a. If yes, how many times a day on average?<br>___________time(s)<br>b. If yes, what type(s) of snacks (i.e., eating in between meals) is/are **<u>usually offered</u>**? (Select all that apply)<br>□ Bars (e.g., energy bars, granola bars, cereal bars) *<br>□ Bread/ sandwiches *<br>□ Cereal (e.g., breakfast cereal, oatmeal) *<br>□ Chips/ salty snacks (e.g., pretzels, popcorn)<br>□ Dairy products/ alternatives (e.g., cow's milk, yogurt, cheese, soy milk) *<br>□ Deep-fried snacks (e.g., French fries, chicken nuggets, doughnuts)<br>□ Frozen dessert (e.g., ice cream, frozen custard)<br>□ Fruits (fresh/ no sugar added dried fruit)/ unsweetened 100% Fruit juice*<br>□ Jerky<br>□ Noodles*

- ☐ Salad/ vegetables*
- ☐ Seed, nuts and legumes (e.g., pumpkin seeds, walnuts, chickpeas) *
- ☐ Soda crackers/ high-fiber wheat biscuits*
- ☐ Sugary drinks (e.g., flavored milk, lactic acid drinks, soft drinks, sports drinks, sweetened fruit juice)
- ☐ Sweet or savory baked pastries (e.g., brownies, cakes, cookies, pie, sausage rolls)
- ☐ Sweets/ chocolates
- ☐ Others, please specify: ______________________________

* A respondent only selects the healthy foods (asterisk items) indicating that a child has a low consumption of unhealthy snacks that eat between meals. In this case, it is scored as "yes=1".

1.5 In the past 7 days, how often did your child consume the following unhealthy snacks and sugar-sweetened beverages?

	Never	1-2 times per week	3-4 times per week	5-6 times per week	Daily
a. Packaged potato chips/ salty snacks (e.g., pretzels, popcorn)					
b. Deep-fried snacks (e.g., French fries, hash browns, fried chicken nuggets, doughnuts)					
c. Frozen dessert (e.g., ice cream, ice pop, milkshake)					
d. Sweet or savory baked pastries (e.g., brownies, cakes, cookies, pie, sausage rolls)					
e. Sweets or chocolate					
f. Sugar-sweetened beverages (e.g., flavored milk, lactic acid drinks, soft drinks, sports drinks, sweetened fruit juice)					

* A respondent answered less than three times a week for the items (a-e) indicating a child has a low consumption of unhealthy snacks. In this case, it is scored as "yes=1". A respondent answered less than three times a week for the items (f) indicating a child has low a consumption of unhealthy beverages. In this case, it is scored as "yes=1".

Additional Questions for assessing children's dietary patterns

1.6	In the past 7 days, how many days did your child eat breakfast?<br>___________day(s)
1.7	In the past 7 days, did your child drink infant formula or follow-up formula milk?<br>□ Yes □ No<br>a. If yes, how many days did your child drink formula milk in the past 7 days?<br>___________day(s)<br>b. Does your child use a feeding bottle to drink formula milk?<br>□ Yes □ No
1.8	In the past 7 days, on average how many glasses of fluid* did your child drink each day?<br>**<u>* Types of fluid included: water, beverages (non-caloric or sugar-sweetened), fruit juices, milk/ alternative (e.g., Plant-based milk) and soup</u>**<br>**<u>1 glass of fluid = 240 mL/ 8 oz</u>**<br>**Portion size guide of 1 glass of fluid (240 mL/ 8 oz):**<br>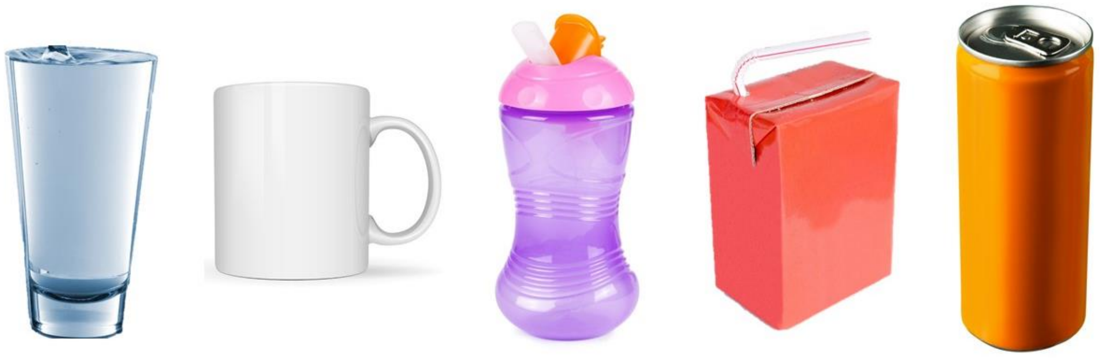<br>**An average of _____________glass (es) per day**<br>Your answer can be in whole or half glasses (e.g., 0.5, 1, 1.5 etc.)<br>* A respondent answered ≥5.5 glasses/day for children aged 2-3-year-old and ≥6.5 glasses/day for children aged 4-8-year-old indicating a child has an adequate intake of water daily. In this case, it is scored as "yes=1".

Part 2: Understanding your child's mealtime behaviours

2. In the past month, how often did the following behaviors occur in your child during meals? (*Reversed item)						
		Never	Rarely	Some-times	Often	Always
**During meals, my child……**						
2.1	Is a picky eater (e.g., refusing to eat a few particular foods/ only being willing to eat certain foods/refusing to try new foods).*	1	2	3	4	5
2.2	Eats very slowly.*	1	2	3	4	5
2.3	Is unable to stay seated at the table.*	1	2	3	4	5
2.4	Refuses to eat meals.*	1	2	3	4	5
2.5	Requires parents to feed him/her to finish a meal.*	1	2	3	4	5
2.6	Eats very small amounts of food.*	1	2	3	4	5
2.7	Throws a tantrum.*	1	2	3	4	5

Part 3: Understanding parents' food choices and preparation for children

3. In the past month, how often did the following behaviors occur when you choose foods and prepare meals for your child?						
		Never	Rarely	Some-times	Often	Always
**For my child, I……**						
3.1	Choose low-fat/ fat-free foods and beverages.	1	2	3	4	5
3.2	Choose low-sodium (salt)/ sodium-free foods and beverages.	1	2	3	4	5
3.3	Cut off visible fat or skin on meat before/ after cooking foods.	1	2	3	4	5

3.4	Choose low-sugar/ sugar-free foods and beverages.	1	2	3	4	5
3.5	Avoid cooking processed meats (e.g., ham, sausages, bacon, corned beef, and biltong/ beef jerky).	1	2	3	4	5
3.6	Choose high-fiber foods and beverages.	1	2	3	4	5
3.7	Choose whole grain versions of foods (e.g., brown rice, wholemeal bread, wheat crackers) instead of refined grains (e.g., white bread and white rice).	1	2	3	4	5
3.8	Avoid cooking foods by deep-frying.	1	2	3	4	5
3.9	Choose to use natural herbs and spices and reduce the amount of high-fat/ -sodium/ -sugar condiments added to foods (e.g., ketchup, barbeque sauce, soy sauces, Nutella).	1	2	3	4	5

Part 4: Understanding your home availability and accessibility of foods

4. In the past 7 days, how often were there available/ accessible <u>(a) Vegetables, (b) Fruits, (c) Unhealthy snacks, (d) Sugar-sweetened beverages, and (e) Plain drinking water</u> in your household?

**In the 7 past days, ……**	Never	Rarely	Some-times	Often	Always
**<u>1. Vegetables …</u>**					
a. were available in our home.	1	2	3	4	5
b. were put where it is easy for my child to get them (e.g., countertop, dining table) at home.	1	2	3	4	5
**<u>2. Fruits …</u>**					
a. were available in our home.	1	2	3	4	5

b. were put where it is easy for my child to get them (e.g., countertop, dining table) at home.	1	2	3	4	5
**3. Plain drinking water …**					
a. were available in our home.	1	2	3	4	5
b. were put where it is easy for my child to get them (e.g., countertop, dining table) at home.	1	2	3	4	5
**4. Unhealthy snacks (e.g., Packaged potato chips/ salty snacks, deep-fried snacks, frozen dessert, sweet or savory baked pastries, sweets or chocolate) …**					
a. were available in our home.	1	2	3	4	5
b. were put where it is easy for my child to get them (e.g., countertop, dining table) at home.	1	2	3	4	5
**5. Sugar-sweetened beverages (e.g., flavored milk, lactic acid drinks, soft drinks, sports drinks, sweetened fruit juice) …**					
a. were available in our home.	1	2	3	4	5
b. were put where it is easy for my child to get them (e.g., countertop, dining table) at home.	1	2	3	4	5

Part 5: Understanding your child's family mealtime environment

5. In the past month, how often did the following mealtime situations occur in your household? (*Reversed item)						
		Never	Rarely	Some-times	Often	Always
5.1	Our family watches TV while eating meals.*	1	2	3	4	5
5.2	Our family eats meals at a routine time.	1	2	3	4	5
5.3	I allow my child to use a phone or tablet during meals.*	1	2	3	4	5
5.4	I let my child eat the same meals as the rest of the family.	1	2	3	4	5
5.5	Our family eats home-cooked meals.	1	2	3	4	5

5.6	I allow my child to play with toys at the dinner table during meals.*	1	2	3	4	5
5.7	Our family eats meals at the dining table (rather than eating in the car, etc.).	1	2	3	4	5
5.8	I let my child eat meals with the rest of the family.	1	2	3	4	5

Appendix B (online supplementary material)

<u>The Assessment Scales of the International Healthy Eating Report Card for Preschool-Aged Children (Chinese Version)</u>

<u>國際學前兒童健康飲食成績表之評估量表</u>

第一部份：了解您孩子的飲食模式

Item		
1.1	在過去 7 天裡，孩子有多少天進食了以下類別的食物？	
	a. 穀物類<br>穀物 (由玉米、米、小麥、或其他穀物製成的食物 (如：麵包、麵條、粥、穀物早餐或其他穀物產品))，以及白色根莖類 (如：薯仔、淮山、木薯) ，或其製成的食物)<br>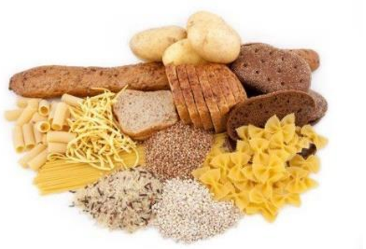	______天
	b. 蔬菜<br>綠葉蔬菜 (如：菠菜、生菜、海帶) 和其他蔬菜 (如：洋蔥、茄子、甜椒、蘑菇、番茄) ，<u>不包括根莖澱粉類蔬菜 (如：薯仔、蕃薯) 及豆類 (如：扁豆、豆)</u><br>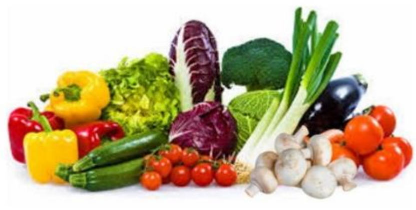	______天
	c. 新鮮水果<br>如：蘋果、哈密瓜、士多啤梨、提子、西瓜、李子 (布冧) 等<br>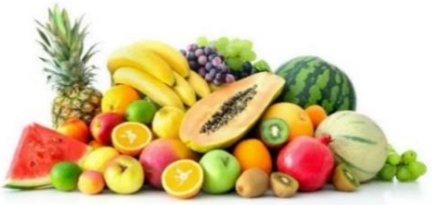	______天

	**d. 肉、家禽、魚、蛋、豆類、堅果和種籽**<br>如：牛肉、豬肉、雞肉、魚、蝦、蟹、雞蛋、青豆、扁豆、豆腐、杏仁、奇亞籽、南瓜子	______天
	**e. 乳製品及代替品**<br>如：牛奶, 芝士, 乳酪, 加鈣豆奶	______天
	* A respondent answered 7 days per week for each food group indicating that a child eats a variety of foods from each of the five main food groups daily. In this case, it is scored as “yes=1”.	
1.2	在過去 7 天裡，孩子平均一天吃多少碗蔬菜？<br>如：綠葉蔬菜 (如：菠菜、生菜、海帶) 或其他蔬菜 (如：洋蔥、茄子、甜椒、蘑菇、番茄) ，**不包括根莖澱粉類蔬菜 (如：薯仔、蕃薯) 及豆類 (如：扁豆、豆)**<br>一碗蔬菜的份量相等於：<br>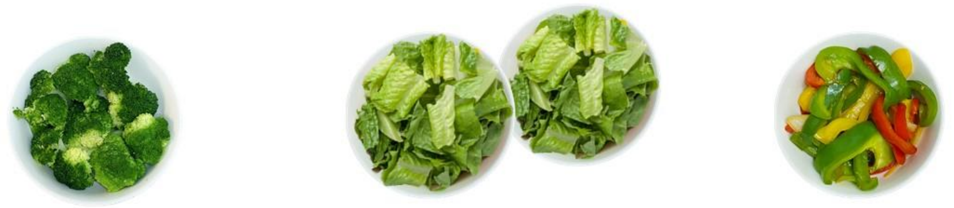<br>= 一碗已煮熟的綠色蔬菜 | = 兩碗生吃的綠葉蔬菜 | = 一碗其他蔬菜 (已煮熟) (如：菇、洋蔥、甜椒、蕃茄、青瓜)<br>*1 碗 = ~ 250 毫升(mL)<br>**平均一天____________碗**<br>您的答案可以是一整碗或半碗 (例如 0.5、1、1.5 等)<br>* A respondent answered ≥1 cup/day for children aged 2-3-year-old or ≥1.5 cup/day for children aged 4-8-year-old indicating that a child has an adequate intake of vegetable daily. In this case, it is scored as “yes=1”.	

1.3	在過去 7 天裡，孩子平均一天吃多少碗新鮮水果？<br><br>**<u>一碗新鮮水果的份量相等於：</u>**<br><br>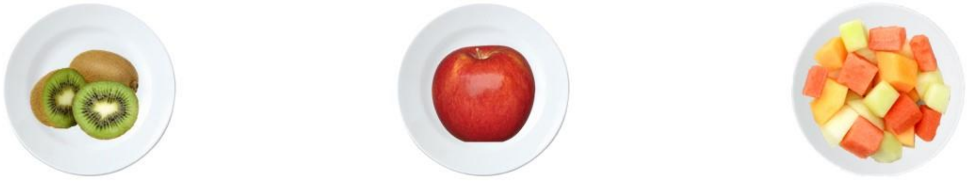<br><br>= 2個小型水果 (例如：奇異果、布冧、小蜜橘、杏桃)<br>= 1個中型水果 (例如：蘋果、香蕉、梨、橙、桃、葡萄柚)<br>= 1碗水果塊 (例如：提子、蜜瓜、芒果、菠蘿)<br><br>*1 碗 = ~ 250 毫升(mL)<br><br>**平均一天____________碗**<br><br>您的答案可以是一整碗或半碗 (例如 0.5、1、1.5 等)<br><br>* A respondent answered ≥1 cup/day for children aged 2-3-year-old or ≥1.5 cup/day for children aged 4-8-year-old indicating that a child has an adequate intake of fruit daily. In this case, it is scored as “yes=1”.
1.4	在過去 7 天裡，孩子有沒有在正餐之間進食茶點？(孩子在幼稚園的情況亦需計算在內。)<br><br>☐ 有 ☐ 沒有<br><br>a. 如有，平均一天吃多少次？<br><br>_________次<br><br>b. 如有，<u>通常</u>給孩子甚麼類型的茶點？ (可選多於一項)<br><br>☐ 小食棒 (如：能量棒、穀物棒) *<br>☐ 麵包/ 三文治*<br>☐ 穀麥片 (如：早餐穀物、燕麥片) *<br>☐ 薯片 / 鹹味零食 (如：百力滋、爆谷)<br>☐ 乳製品/ 代替品(如：牛奶、乳酪、芝士、豆奶)*<br>☐ 油炸食物 (如：炸薯條、炸雞塊、冬甩)<br>☐ 冷凍甜品 (如：雪糕、奶凍)<br>☐ 水果(新鮮/無加糖乾果)/ 無加糖的 100% 果汁*<br>☐ 肉乾<br>☐ 麵*

	□ 沙律/ 蔬菜*<br>□ 種子、堅果和豆類 (如：南瓜子、核桃、鷹嘴豆) *<br>□ 蘇打餅/ 高纖維小麥餅乾*<br>□ 含糖飲品 (如：有味奶、乳酸類飲品、汽水、運動能量飲品、加糖果汁飲品)<br>□ 鹹甜烘焙糕點 (如：布朗尼、蛋糕、曲奇餅、批、香腸捲)<br>□ 糖果/ 巧克力<br>□ 其他，請註明： ____<br>* A respondent only selects the healthy foods (asterisk items) indicating that a child has a low consumption of unhealthy snacks that eat between meals. In this case, it is scored as “yes=1”.

1.5 在過去 7 天裡，孩子有幾經常會進食/喝以下不健康零食或含糖飲品？

	從不	每週<br>1-2 次	每週<br>3-4 次	每週<br>5-6 次	每天
a. 包裝薯片/鹹味零食 (如：百力滋、爆谷)					
b. 油炸小食 (如：炸薯條、炸薯餅、炸雞塊、冬甩)					
c. 冷凍甜點 (如：雪糕、雪條、奶昔)					
d. 鹹甜烘焙糕點 (如：布朗尼、蛋糕、曲奇餅、批、香腸捲)					
e. 糖果或朱古力					
f. 含糖飲品 (如：有味奶、乳酸類飲品、汽水、運動能量飲品、加糖果汁飲品)					

* A respondent answered less than three times a week for the items (a-e) indicating a child has a low consumption of unhealthy snacks. In this case, it is scored as “yes=1”. A respondent answered less than three times a week for the items (f) indicating a child has low a consumption of unhealthy beverages. In this case, it is scored as “yes=1”.

Additional Questions for assessing children's dietary patterns

1.6	在過去 7 天裡，孩子有多少天吃早餐？<br>_________天
1.7	在過去 7 天裡，孩子有沒有喝配方奶粉或較大嬰兒/幼兒配方奶粉（俗稱「大仔奶粉」）？<br>☐ 有 ☐ 沒有<br>a. 如有，在過去 7 天裡，孩子有多少天喝配方奶粉？<br>_________天<br>b. 孩子有沒有使用奶樽喝配方奶？<br>☐ 有 ☐ 沒有
1.8	在過去 7 天裡，孩子平均一天喝多少杯流質飲品*？<br>**<u>* 流質飲品類型包括：水、飲品 (不含熱量/ 含糖) 、果汁、牛奶/ 替代品 (如：植物奶) 及湯</u>**<br><u>1 杯流質飲品= 240 毫升(mL)/ 8 盎司(oz)</u><br>1 杯流質飲品(240 毫升/ 8 盎司)的份量示意圖:<br>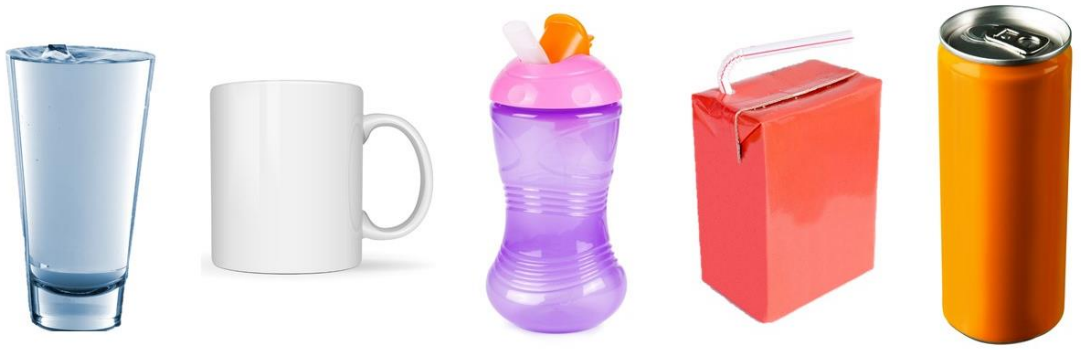<br>平均一天__________杯<br>您的答案可以是一整杯或半杯 (例如 0.5、1、1.5 等)<br>* A respondent answered ≥5.5 glasses/day for children aged 2-3-year-old and ≥6.5 glasses/day for children aged 4-8-year-old indicating a child has an adequate intake of water daily. In this case, it is scored as "yes=1".

第二部份：了解孩子進食時的行為

2. 在過去一個月裡，孩子有幾經常在用餐期間出現以下行為？ (*Reversed item)						
		從不	甚少	有時	經常	總是
<u>**在用餐期間，我的孩子……**</u>						
2.1	是偏食者 (如：經常拒絕吃/ 只願吃某些食物/ 拒絕嘗試新食物)。*	1	2	3	4	5
2.2	吃得很慢。*	1	2	3	4	5
2.3	不能在餐桌前「坐定定」。*	1	2	3	4	5
2.4	拒絕吃飯。*	1	2	3	4	5
2.5	需要父母餵食才完成用餐。*	1	2	3	4	5
2.6	進食的份量非常少。*	1	2	3	4	5
2.7	發脾氣。*	1	2	3	4	5

第三部份： 了解家長為孩子的食物選擇及準備

3. 在過去一個月裡，為您的孩子選擇食物和準備飯餸時，您有幾經常做出以下句子所描述的行為?						
		從不	甚少	有時	經常	總是
<u>**為我的孩子，我……**</u>						
3.1	選擇低脂/脫脂的食物及飲品。	1	2	3	4	5
3.2	選擇低鈉 (鹽) /無鈉的食物及飲品。	1	2	3	4	5
3.3	在烹飪前後會去除肉類中可見的脂肪/皮。	1	2	3	4	5
3.4	選擇低糖/無糖的食物及飲品。	1	2	3	4	5
3.5	避免烹調加工肉類製品 (如：火腿、腸仔、煙肉、午餐肉、鹹牛肉)。	1	2	3	4	5

3.6	選擇高纖維的食物及飲品。	1	2	3	4	5
3.7	選擇全穀物食品 (如：糙米、全麥麵包、麥餅) ，而不是精製穀物 (如：白麵包、白米)。	1	2	3	4	5
3.8	避免使用油炸方式烹調食物。	1	2	3	4	5
3.9	選擇用天然調味品調味，減少在食物中加入高油/高鈉/高糖的調味醬料 (如：番茄醬、燒烤醬、蠔油、榛子巧克力醬)。	1	2	3	4	5

第四部份： 了解您家中食物的可獲得性及可及性

4. 在過去 7 天裡， (a) 蔬菜、 (b) 水果、 (c) 不健康零食、 (d) 含糖飲品及 (e) 飲用水有幾經常在您的家中找到及擺放在讓孩子能方便拿取的位置？

**在過去 7 天裡，**	從不	甚少	有時	經常	總是
<u>1. 蔬菜…</u>					
a. 可在家中找到的。	1	2	3	4	5
b. 擺放在家中讓孩子能方便拿取的位置 (如：廚房灶頭枱面、餐桌) 。	1	2	3	4	5
<u>2. 水果…</u>					
a. 可在家中找到的。	1	2	3	4	5
b. 擺放在家中讓孩子能方便拿取的位置 (如：廚房灶頭枱面、餐桌) 。	1	2	3	4	5
<u>3. 飲用水 …</u>					
a. 可在家中找到的。	1	2	3	4	5
b. 擺放在家中讓孩子能方便拿取的位置 (如：廚房灶頭枱面、餐桌) 。	1	2	3	4	5

4. 不健康零食 (如：包裝薯片/鹹味零食、油炸小食、冷凍甜點、鹹甜烘焙糕點、糖果或朱古力) …					
a. 可在家中找到的。	1	2	3	4	5
b. 擺放在家中讓孩子能方便拿取的位置 (如：廚房灶頭枱面、餐桌) 。	1	2	3	4	5
5. 含糖飲品 (如：有味奶、乳酸類飲品、汽水、運動能量飲品、加糖果汁飲品) …					
a. 可在家中找到的。	1	2	3	4	5
b. 擺放在家中讓孩子能方便拿取的位置 (如：廚房灶頭枱面、餐桌) 。	1	2	3	4	5

第五部份：了解您孩子的家庭用餐時的環境

5. 在過去一個月裡，您們家有幾經常出現以下句子所描述的用餐情況？(*Reversed item)						
		從不	甚少	有時	經常	總是
5.1	我們一邊看電視一邊吃飯。*	1	2	3	4	5
5.2	我們家在固定的時間吃飯。	1	2	3	4	5
5.3	我允許我的孩子在用餐時使用手機或平板電腦。*	1	2	3	4	5
5.4	我會讓我的孩子吃與家人一樣的飯菜。	1	2	3	4	5
5.5	我們家吃住家餸菜。	1	2	3	4	5
5.6	我允許我的孩子在用餐時在餐桌上玩玩具。*	1	2	3	4	5
5.7	我們一家人在餐桌上吃飯 (而不是在車上吃等等) 。	1	2	3	4	5
5.8	我會讓我的孩子與家人一起吃飯。	1	2	3	4	5

Appendix C (online supplementary material)

Items corresponding to the latent variable

Latent variable	Item	Example of wording	Scale
**Scale of children's eating behaviours in IHERCS**			
Food variety	1.1, 1.2, 1.3	*In the past 7 days, on average, how many cups of vegetables did your child eat each day?*	Open-ended questions (i.e., answering the number of days and the amount consumed)
Low consumption of unhealthy foods	1.4, 1.5(a-e), 1.5(f)	*In the past 7 days, how often did your child consume the following unhealthy snacks and sugar-sweetened beverages? (e.g., Packaged potato chips/ salty snacks (e.g., pretzels, popcorn))*	5-point Likert scales (i.e., ranging from Never to Daily), and Multiple choices (i.e., responding to a list of snack and drink items)
Mealtime behaviours	2.1-2.7	*Eats very slowly.** *(Following the stem of "My child …".)*	5-point Likert scale (ranging from Never to Always)
**Scale of family home food environments in IHERCS**			
Parental food choices	3.1, 3.2, 3.4, 3.6, 3.7, 3.9	*Choose low-fat/ fat-free foods and beverages.* *(Following the stem of "For my child, I …".)*	5-point Likert scale (ranging from Never to Always)
Parental food preparation	3.3, 3.5, 3.8	*Avoid cooking foods by deep-frying.* *(Following the stem of "For my child, I …".)*	5-point Likert scale (ranging from Never to Always)

Availability of healthy food	4.1.a, 4.2.a, 4.3.a	*Vegetables were available in our home.*<br>*(Following the stem of "In the 7 past days, …".)*	5-point Likert scale (ranging from Never to Always)
Availability of unhealthy food	4.4.a, 4.5.a	*Unhealthy snacks were available in our home.*<br>*(Following the stem of "In the 7 past days, …".)*	5-point Likert scale (ranging from Never to Always)
Accessibility of healthy food	4.1.b, 4.2.b, 4.3.b	*Vegetables were put where it is easy for my child to get them (e.g., countertop, dining table) at home.*<br>*(Following the stem of "In the 7 past days, …".)*	5-point Likert scale (ranging from Never to Always)
Accessibility of unhealthy food	4.4.b, 4.5.b	*Unhealthy snacks were put where it is easy for my child to get them (e.g., countertop, dining table) at home.*<br>*(Following the stem of "In the 7 past days, …".)*	5-point Likert scale (ranging from Never to Always)
Reduction in Mealtime distraction	5.1, 5.3, 5.6	*Our family watches TV while eating meals.**	5-point Likert scale (ranging from Never to Always)
Structure of family mealtime	5.2, 5.4, 5.5, 5.7, 5.8	*Our family eats meals at a routine time.*	5-point Likert scale (ranging from Never to Always)

Note. Revised items are marked with an asterisk (*).
